# Efficiency of Cytology Samples for PD-L1 Evaluation and Comparison with Tissue Samples*

**DOI:** 10.5146/tjpath.2020.01494

**Published:** 2020-09-15

**Authors:** İbrahim Kulaç, Aslı Aydın, Pınar Bulutay, Pınar Fırat

**Affiliations:** Department of Pathology, Koç University School of Medicine, İstanbul, Turkey; Department of Medical Student, Koç University School of Medicine, İstanbul, Turkey

**Keywords:** PD-L1, Cytology, Cell blocks

## Abstract

***Objective:*** Lung cancer is the leading cause of cancer-related death. PD-L1 blockers have become a first-line option for advanced non-small cell lung cancer (NSCLC) patients. Guidelines require the assessment of PD-L1 expression by immunohistochemistry. Although tissue samples are widely used, cytologic samples could be an alternative. In this study, we compared cytologic samples with tissue samples for PD-L1 evaluation in NSCLC cases.

***Material and Method:*** Koç University Hospital, Department of Pathology Laboratory Information System was scanned for all PD-L1 tests performed on NSCLC cases, either on tissue samples or cell blocks. The type of the biopsy/aspiration procedure, the tumor type, patient demographics, and the percentage of PD-L1 positive tumor cells were recorded. A total of 73 tissue samples and 49 cell blocks were found to be eligible for the study.

***Results:*** The PD-L1 positivity score was at least 1% in 44 of 73 samples of the tissue group and 19 of 49 samples of the cell block group. Tissue samples showed significantly higher positivity compared to the cell blocks (p=0.020). Comparing the frequency of cases with ≥50% positivity showed no statistically significant difference. A comparison of PD-L1 positivity rates of only the small biopsies and cell blocks also showed no significant difference.

***Conclusion:*** Although they harbor a limited number of tumor cells, cell blocks prepared from cytologic samples are good alternatives for PD-L1 testing. However, large resections should be used for PD-L1 evaluation whenever possible since even 1% positivity may affect the treatment decision.

## INTRODUCTION

Lung cancer is the leading cause of cancer-related death worldwide (1). The majority of lung cancer cases are non-small cell carcinoma (NSCLC). Along with classical treatment modalities for NSCLC (surgical resection, chemotherapy/radiotherapy…), targeted therapeutics have already become a part of routine oncology practice (2). Elucidating the molecular pathogenesis of NSCLC has led to the discovery of a number of driver mutations, especially in various receptor tyrosine kinases (3) which are now among the most popular targets for metastatic NSCLC treatment. Along with tyrosine kinase inhibitors, immune checkpoint inhibitors (PD1 and PD-L1 blockers, CTLA4 inhibitors) have changed the fate of the patient with lung cancer (4).

Programmed cell death 1 (PD1) is a molecule expressed on the surface of T cells that have an immunoregulatory function. The interaction of PD1 and its ligand PD-L1 prevents host cells from the immune response, and this interaction therefore functions as an inhibitory mechanism against potential autoimmune reactions (5). It has been shown that tumor cells also avoid immune destruction by expressing PD-L1. Monoclonal antibodies developed against PD-L1 (and PD1) block the PD-L1-PD1 interaction and inhibit antitumoral activity, which then activates the cytotoxic immune response against tumor cells (6).

A significant percentage of lung cancers are advanced at the time of diagnosis and PD-L1 blockers have become a first line treatment option for these patients (7). It is very well known that PD-L1 inhibitors are more effective in tumors that have higher PD-L1 expression. Current guidelines therefore require the determination of PD-L1 expression levels of a given tumor by immunohistochemistry, and the cut-off points for certain immunotherapeutic drugs are clearly defined (7).

In general, biopsy (tissue) specimens are used for immunohistochemical studies to determine the level of PD-L1 expression. However, cell blocks constructed from cytologic specimens such as transthoracic or endobronchial aspirations as well as serous effusions are also useful alternatives. We hypothesized that cytology samples would be as representative as tissue samples for the evaluation of PD-L1 expression.

## MATERIAL and METHODS

The Koc University Hospital, Department of Pathology Laboratory Information System was scanned for all PD-L1 tests performed on NSCLC cases between August 2018-December 2019, either on tissue samples or cell blocks. The type of the biopsy/aspiration procedure, the tumor type, patient demographics, and percentage of PD-L1 positive tumor cells (positive tumor cells/all tumor cells*100) were recorded. In total, 73 tissue samples and 49 cell blocks were found to be eligible for the study.

### Sample Preparation

Aspiration samples are first sprayed on glass slides and fixed immediately with a few drops of absolute ethanol. After waiting 10 seconds, clumps are transferred into a container and fixed for 24 hours in 10% formalin. Fixed samples are then processed through the routine processing protocol.

Effusion samples are centrifuged, and the supernatant is tossed away. The sediment is placed on a slide and mixed with 4-5 drops of plasma and 4-5 drops of thromboplastin. The sample is centrifuged again and topped with 10% formalin. The clumped sample is then placed into a cassette and processed through the routine processing protocol.

Tissue samples are fixed in formalin for 24 hours and processed using the routine processing protocol.

### PD-L1 Immunohistochemistry

For immunohistochemistry, 3 µm thick sections were obtained from the representative paraffin blocks. A validated protocol for anti-PD-L1 antibody (Ventana, Clone: SP63) was followed on the Ventana Benchmark XT Autostainer (8). PD-L1 was evaluated if the sample had more than 100 tumor cells. Samples with fewer than 100 cells were excluded from the study.

### Statistical Analysis

Numeric variables were analyzed by their mean and minimum-maximum values, while categorical variables were included in the analysis by numbers and percentages. The Chi-Square test was used for the comparison of categorical data and the Mann-Whitney U (Wilcoxon rank-sum) test for continuous data. The Spearman correlation coefficient was used for the comparison of two numeric variables. The statistical significance threshold was accepted as P<0.05. The Stata V13 software was used for statistical analyses.

This study was approved by the Koç University Ethics Committee with IRB approval number 2019.407.IRB2.128

## RESULTS

A total of 122 samples were eligible for the study, consisting of 73 tissue samples and 49 cell blocks prepared from aspiration/effusion materials. The majority of the cases were adenocarcinomas. There were 75 males and 47 females. Detailed demographic information is summarized in Table 1.

**Table 1:** Distribution of the age and sex of the patients

	**Cell Block**	**Tissue**	**Overall**
Age (min/max/mean)	44/88/63.9	20/86/63.8	20/88/63.89
Sex (M/F)	32/17	43/30	75/47

### Tissue Samples

All cases were diagnosed as non-small cell lung cancer or had non-small cell component in the tumor mass. There were 47 adenocarcinomas and 12 squamous cell carcinomas (the distribution of the diagnosis of the rest of the cases is detailed in Table 2).

**Table 2:** Distribution of the tumor types of all cases

**Tumor Type**	**Cell Block**	**Tissue**	**Total**
Adenocarcinoma	27	47	74
High Grade Neuroendocrine Tumor	0	1	1
Carcinosarcoma	0	2	2
NSCLC	15	6	21
Pleomorphic Carcinoma	0	3	3
Squamous Cell Carcinoma	7	12	19
Sarcomatoid Carcinoma	0	1	1
Small Cell Carcinoma + Adenocarcinoma	0	1	1
Total	**49**	**73**	**122**

### Cell Blocks

All cases were diagnosed as non-small cell lung cancer; 27 were adenocarcinoma and 7 were squamous cell carcinoma (the distribution of the diagnosis of the rest of the cases is detailed in Table 2).

**Figure 1:**
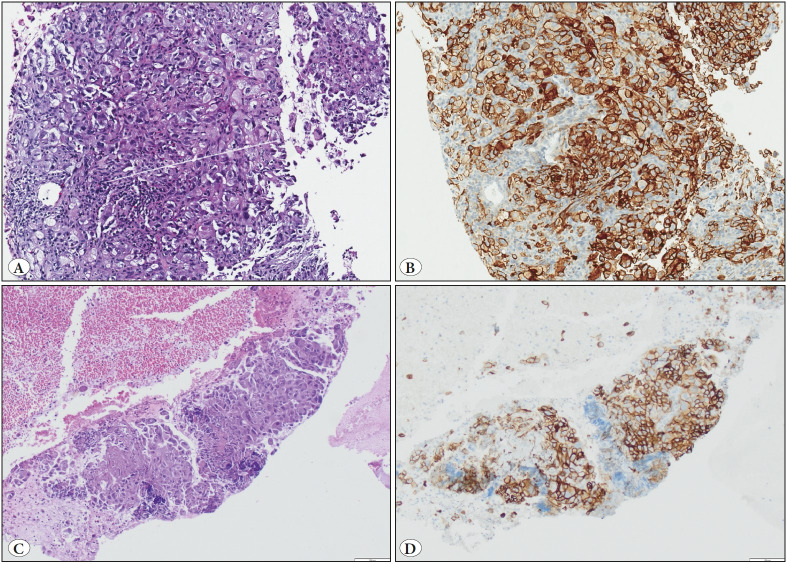
**A-B)** A lung biopsy sample showing 90% PDL1 positivity (H&E; x100). **C-D)** A cell block sample prepared from the aspiration cytology material of a metastatic lung adenocarcinoma showing high PDL1 positivity (IHC; x100).

The PD-L1 positivity score was at least 1% in 44 of the 73 tissue samples and 19 of the 49 cell block samples (representative PD-L1 staining of a tissue and a cell block sample are demonstrated in Figure 1 and the PD-L1 scores of the samples are displayed in Table 3). Overall comparison of positivity rates (all samples were categorized into two groups: positive or negative, in which staining in ≥1% of the tumor cells was considered as positive and staining in <1% of the tumor cells was considered as negative) showed a statistically significant difference between two sample types; biopsy/resection specimens showed a significantly higher positivity rate compared to the cell blocks (chi-square, p=0.020). Comparing the frequency of cases with ≥50% positivity rate showed no statistically significant difference between the groups (chi-square, p>0.05).

**Table 3:** Distribution of the samples in groups assigned by PD-L1 scores of <1%, 1-50%, ≥50%.

	**PD-L1 Score**			
	**Negative**	**Positive**		
**Sample Type**	**<1%**	**1-50%**	**≥50%**	**Total**
Cell Block (n)	30	9	10	49
Tissue (n)	29	28	16	73
Total	59	37	26	122

A comparison of PD-L1 positivity rates of only the small biopsies (tru-cut, bronchoscopic, mediastinoscopic) and cell blocks also showed no significant difference (chi-square, p>0.05). A rank sum analysis of the positivity scores of the two sample types showed no statistically significant difference (Mann-Whitney U test, p>0.05).

## DISCUSSION

PD-L1 blockers have been a very effective treatment option for advanced NSCLC patients. Expression of PD-L1 as evaluated by immunohistochemistry (IHC) is required for anti-PD-L1 treatment. There are a number of antibodies that can be used, and the score is given by the percentage of PD-L1 positive tumor cells among all tumor cells on a given slide. Tissue samples are widely used and have been a standard sample for evaluation of PD-L1 positivity. In this study, we aimed to compare cytologic samples with tissue samples.

Obtaining cell blocks by cytologic sampling such as endobronchial ultrasound-guided aspiration of mediastinal lymph nodes or centrally located lesions, or by the aspiration of serous effusion, is less invasive compared to trans-thoracic tru-cut biopsies, open biopsies or resections. These samples are often adequate for the final diagnosis as well as for molecular tests (EGFR mutation analysis…). However, the samples may also be used for PD-L1 IHC if they contain a sufficient number of tumor cells.

We compared 49 cytologic samples with 73 tissue samples and looked for differences in PD-L1 positivity rates and scores in this study. Although tissue samples had a higher rate of positive PD-L1 results, the rates of >50% positivity did not show a statistically significant difference. Given the fact that tru-cut biopsies and mediastinoscopic biopsies provide a generous amount of material for pathologists for PD-L1 evaluation although they contain a limited number of tumor cells, we compared tru-cut biopsies and mediastinoscopic biopsies with cell blocks and found no statistically significant difference.

It is known that PD-L1 expression may vary in a single tumor mass (9). It is best to evaluate as many cells as possible from a tumor mass. However, pathologists often have to deal with small biopsy samples or cytologic specimens due to the high rate of unresectable NSCLC cases. A few publications pointed out the problems of small biopsies and cytological samples for PD-L1 evaluation (10). The concordance figures of small biopsies and resection specimens vary between 52% and 92% (11,12). This may be due to intra-tumoral heterogeneity, tissue handling, and scoring algorithms, as well as interobserver variability. There are also a few studies on the effectiveness of cytologic samples (either cell blocks or smears) for the evaluation of PD-L1 expression in NSCLC. One of the studies had results that were similar to the current study, as tumors with higher score of PD-L1 positivity had a higher concordance in paired cytologic and resection samples (13). Despite all these, the PD-L1 scores showed fairly acceptable concordance between cytological and biopsy samples, as shown by multiple studies (14–16).

**Figure 2:**
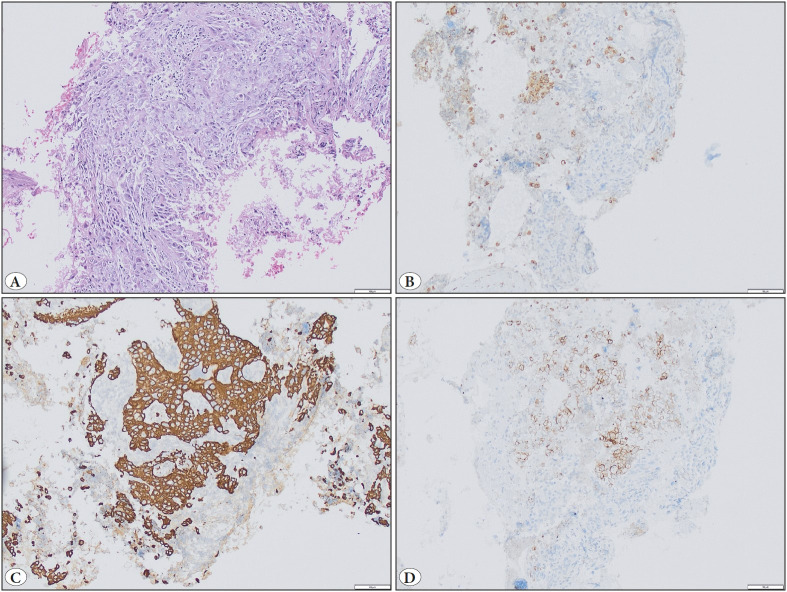
PDL1 evaluation of a cell block sample rich in histiocytes. **A)** Tumor cells and accompanying histiocytes (H&E; x100). **B)** CD68 staining highlights the presence of plenty of histiocytes (IHC; x100). **C)** Pancytokeratin shows epithelial tumor cells (IHC; x100). **D)** PDL1 is positive in tumor cells as well as the histiocytes (IHC; x100).

As mentioned elsewhere, PD-L1 evaluation on cell blocks may be difficult due to the positivity on histiocytes (Figure 2) (17). Although one can encounter this problem with any type of sample while evaluating PD-L1 expression, differentiating a tumor cell and a histiocyte could be more difficult with a cell block due to the lack of the tumor architecture as well as the altered morphology. This may result in false positives. We therefore meticulously evaluate PD-L1 on cell blocks during our daily practice, and the consensus of at least two pathologists is written on the final report.

There are two major weaknesses of this study. One is that the samples that were compared are not from the same patients. PD-L1 expression may certainly differ from case to case and an ideal study should compare cytologic and tissue samples from the same patients. This study is also only based on Ventana SP263 staining and we did not look for the concordance of other antibodies that are commercially available.

In conclusion, cell blocks prepared from cytologic samples are good alternatives for PD-L1 testing although they harbor a limited number of tumor cells. It should be kept in mind that large resections should be used for PD-L1 evaluation when possible since even 1% positivity may affect the decision for treating a patient.
